# The correlation between FDG-PET metabolic parameters, PD-L1 expression and p16/HPV status in oropharyngeal cancers

**DOI:** 10.1007/s00405-025-09982-w

**Published:** 2026-01-21

**Authors:** Angéla Horváth, Imre Uri, Emese Kristóf, Gábor Lotz, Éva Kocsmár, Bianka Gurbi, Dávid Keresztes, László Tamás, Gábor Polony, Tamás Györke, Kornél Dános

**Affiliations:** 1 Department of Oto-Rhino-Laryngology, Head and Neck Surgery, Semmelweis University, Szigony Str. 36, Budapest, 1083 Hungary; 2 Medical Imaging Center, Department of Nuclear Medicine, Semmelweis University, Üllői út 78/A, Budapest, 1082 Hungary; 3 Department of Pathology, Forensic and Insurance Medicine, Semmelweis University, Üllői Str. 93, Budapest, 1091 Hungary; 4 Department of Molecular Biology, Semmelweis University, Tűzoltó Str. 37- 47, Budapest, 1094 Hungary

**Keywords:** Oropharyngeal cancers, FDG-PET, Metabolic parameters, PD-L1 expression

## Abstract

**Purpose:**

Immunotherapy targeting the PD-1/PD-L1 axis has emerged as a promising strategy in oropharyngeal squamous cell carcinoma (OPSCC). However, reliable non-invasive biomarkers to predict immune checkpoint expression and treatment response are still lacking. This study aimed to investigate the relationship between PET/CT-derived metabolic parameters, PD-L1 expression, and p16/HPV status, and their prognostic value in OPSCC.

**Methods:**

In this retrospective analysis, 60 patients with newly diagnosed OPSCC who underwent baseline 18F-FDG PET/CT were included. Tumor PD-L1 expression was assessed using both Tumor Proportion Score (TPS) and Combined Positive Score (CPS). Correlations were analyzed between metabolic PET/CT parameters (maximum standardized uptake value [SUVmax], metabolic tumor volume [MTV], and total lesion glycolysis [TLG]) and PD-L1 expression. Correlation analyses, survival outcomes, and response to chemoradiotherapy were evaluated, with subgroup analyses based on p16 and HPV DNA status.

**Results:**

Significant positive correlations were observed between volumetric PET/CT parameters and PD-L1 expression. In HPV DNA-positive OPSCC, PET/CT-derived volumetric parameters (MTV and TLG) showed positive correlations with PD-L1 expression (TPS: rho = 0.501 and 0.512; CPS >20: rho = 0.441 and 0.491). Higher MTV and TLG were associated with significantly shorter overall survival (*p* = 0.005 and *p* = 0.001, respectively). Multivariate Cox regression showed the strong prognostic effect of TLG, independent of p16, MTV and even primary tumor stage. Staging MTV was found to be an independent predictor of complete response to chemoradiotherapy (*p* = 0.026, OR (odds ratio) = 1.08). (No significant correlation was found in the p16-negative subgroup.)

**Conclusions:**

Metabolic tumor burden, reflected by PET/CT-derived MTV and TLG, is positively associated with PD-L1 expression and clinical outcomes in OPSCC. These findings suggest a biologically meaningful link between tumor glycolytic activity and immune checkpoint regulation, particularly in HPV-associated cases. PET/CT-based metabolic profiling may serve as a non-invasive tool for risk stratification and immunotherapy planning in OPSCC.

## Introduction

Oropharyngeal squamous cell carcinoma (OPSCC) is a major subset of head and neck cancers, with a rising global incidence. The disease exhibits two quite different pathways, separated as p16 positive and negative OPSCC according to UICC TNM8. p16 positivity is accepted as a surrogate marker for human papillomavirus (HPV)-associated OPSCC, primarily linked to HPV-16 infection. In contrast, p16 negative (and in most cases HPV-negative) OPSCC is strongly associated with traditional risk factors such as smoking and alcohol consumption causing high mutational tumor burden and field cancerization.

P16-positive OPSCC is generally associated with better prognosis, improved treatment response, and distinct immune microenvironmental features, whereas p16-negative tumors often present with more aggressive behavior and poorer outcomes [1].

Recent Hungarian data from oropharyngeal cancer patients also supports this phenomenon, demonstrating favorable chemoradiotherapy response rates in the p16-positive population [2]. Although, recent studies revealed minor discordance of p16- and HPV positivity, both markers proved independent positive prognostic factors [3, 4].

 18 F-Fluorodeoxyglucose Positron Emission Tomography/Computed Tomography (18F-FDG PET/CT) is widely used for staging, planning, treatment response monitoring in OPSCC. Its prognostic use is currently the subject of numerous studies. In addition to the well-known maximum standardized uptake value (SUVmax), metabolic tumor volume (MTV), and total lesion glycolysis (TLG) are considered as potential prognostic factors. High values of these parameters are associated with worse overall survival (OS) as well as disease-free survival (DFS) [5].

Programmed death-ligand 1 (PD-L1) expression plays a pivotal role in tumor immune evasion and response to immune checkpoint inhibitors such as pembrolizumab [6]. The assessment of PD-L1 status via tumor proportion score (TPS) or combined positive score (CPS) is commonly used to stratify patients for immunotherapy.

The relationship between PD-L1 expression, PET/CT metabolic activity, and p16/HPV status remains poorly understood, necessitating further investigation. This study aims to evaluate metabolic and immune characteristics between p16 positive, p16 and HPV DNA positive and p16 negative OPSCC.

Understanding these relationships may provide valuable prognostic insights and help refine treatment strategies for OPSCC.

## Methods

### Study population

Patients were included with newly diagnosed oropharyngeal cancer between 2017 and 2024 at the Department of Oto-Rhino-Laryngology and Head and Neck Surgery, Semmelweis University, who underwent staging 18F-FDG PET/CT at the Department of Nuclear Medicine. The histological specimens of these patients were subjected to PD-L1 determination and p16 immunohistochemistry at the Department of Pathology, and p16 positive cases were tested to HPV DNA typing by real-time PCR at the Department of Molecular Biology.

Patients were excluded from the study if they had undergone PET/CT imaging at an external institution, if the histopathological diagnoses were not performed at our tertiary referral center, or if they had received any prior oncologic treatment for head and neck malignancy. Further exclusion criteria included a primary tumor size below 2 cm (due to limitations in metabolic parameter accuracy related to partial volume effects), non-evaluable PD-L1 or p16 immunohistochemistry, or if the histological diagnosis was not squamous cell carcinoma. We performed a retrospective cohort study examining the electronic medical records.

### Ethical approval

The study was performed with the ethical permission of Semmelweis University (SE IKEB 105/2014).

### p16, HPV status determination

The presence of p16 overexpression, detected via immunohistochemistry (IHC), is a widely used surrogate marker for HPV infection [7] However, p16-positivity alone does not confirm the presence of viral DNA, so real-time polymerase chain reaction (PCR) was performed to directly detect HPV DNA in p16-positive cases, enhancing diagnostic accuracy [8].

The expression of p16^INK4^ protein was assessed by immunohistochemistry and considered positive when ≥70% of tumor cells showed strong nuclear and cytoplasmic staining [9]. High-risk human papillomavirus DNA detection was performed using a DNA isolation procedure (QIAamp DNA FFPE Advanced Kit) and a DNA-based polymerase chain reaction (PCR) assay (GeneProof Human Papillomavirus (HPV) Screening PCR Kit) capable of identifying 23 high-risk HPV genotypes, including types 16, 18, 26, 30, 31, 33, 34, 35, 39, 45, 51, 52, 53, 56, 58, 59, 66, 67, 68, 69, 70, 73, 82, and 97. The procedure was performed according to the manufacturer’s protocols.

### PD-L1 evaluation

PD-L1 immunohistochemistry was performed using DAKO 22C3 pharmDx Kit (Dako Denmark A/S, Glostrup, Denmark) on a Ventana Benchmark Ultra automated immunostaining system (Ventana Medical Systems, Inc., Tucson, AZ, USA). For this purpose, 4 μm thick tissue sections were freshly cut from the formalin-fixed, paraffin-embedded tissue block of the tested cancer and mounted on coated glass slide (TOMO Adhesion Microscope Slide, Matsunami Glass Ind., Ltd. Kishiwada City, Osaka, Japan) along with a positive control tonsil tissue section. For each case, a separate slide was also prepared with a section of the tested tissue to serve as a negative control.

IHC staining was performed using monoclonal mouse anti-PD-L1 clone 22C3 (and negative control reagent for negative control slides) according to the manufacturer’s recommendations but using OptiView DAB Detection Kit and OptiView Amplification Kit (Ventana Medical Systems, Inc., Tucson, AZ, USA) for signal development, as previously published by Vainer et al. [10] PD-L1 expression was assessed by immunohistochemistry and evaluated using the Tumor Proportion Score (TPS) and Combined Positive Score (CPS). TPS was defined as the percentage of viable tumor cells showing partial or complete membranous PD-L1 staining at 200X magnification (20X objective), regardless of staining intensity. CPS was calculated based on the number of PD-L1–positive tumor cells, lymphocytes, and macrophages, in relation to the total number of viable tumor cells, with the final value capped at 100.

Only cells within viable tumor areas were included in the analysis; necrotic regions and immune cells associated with necrosis were excluded. A minimum of 100 viable tumor cells was required for a sample to be evaluable. For tumor cells, only membranous staining was considered positive, while both membranous and cytoplasmic staining were accepted for immune cells.

Scoring was performed manually by two specially trained pathologists (GL, ÉK), independently. In cases of discrepancy, final scores were determined by joint review and consensus.

### PET/CT imaging and metabolic parameter analysis

Patients received 2.5–3.0 MBq/kg of 18F-Fluorodeoxyglucose (18F-FDG) intravenously following a fasting period of at least 6 h. Sixty minutes post-injection, a low-dose, non-contrast-enhanced CT scan and three-dimensional PET emission images were acquired from the jugulum to the mid-thigh. This was followed by an additional dedicated scan of the head and neck region (from the frontal sinus to the aortic arch) with the arms in the down position. In selected cases, imaging was performed for radiotherapy planning using a dedicated flat scanning table and a thermoplastic head and neck immobilization mask.

All scans were completed on a hybrid PET/CT scanner (GE Discovery IQ5; GE Healthcare, Milwaukee, WI, USA). Image reconstruction was performed using a Bayesian penalized-likelihood reconstruction algorithm with point-spread function modeling (Q.Clear, β = 350). This was the one reconstruction performed similarly for every emission scan, and it also accounts for partial volume correction [11].

**Fig. 1:**
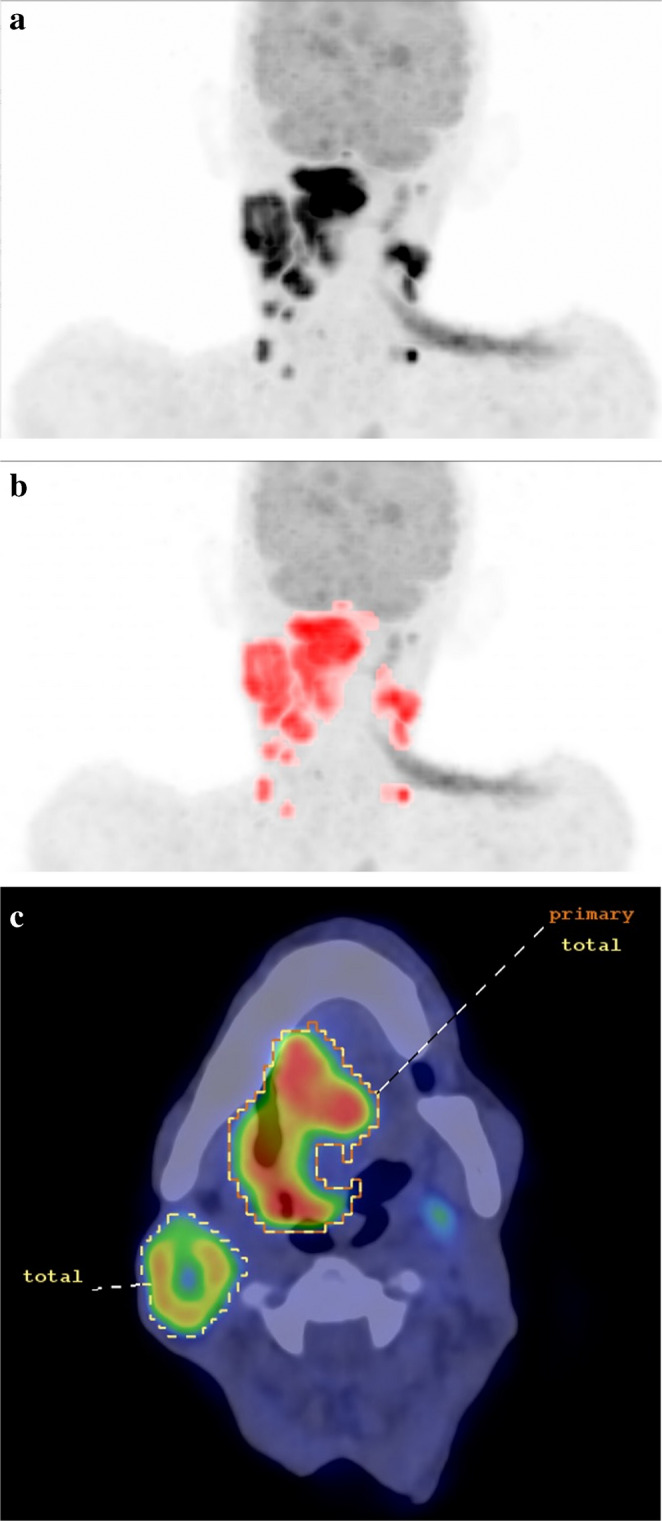
Tumor segmentation procedure. **a**) original three-dimensional PET maximum intensity projection (MIP) image of the head and neck, intensive uptake of the meso-hypopharyngeal squamous cell carcinoma and the bilateral neck lymph node metastases; **b**) automatic segmentation of the total tumor volume with SUV low threshold of 2.5 g/ml (volumes of interest in red), this method was kept as reference, since it could not exclude every region with physiologic or inflammatory uptake close to the tumor; **c**-**d**) semi-automatic segmentation of the primary tumor (orange) and total tumor volume (yellow, primary and metastatic nodes together) using a SUV low threshold of 2.5 g/ml and manual correction if needed according to the anatomical landmarks on CT

PET images were analyzed using Mediso InterView™ Fusion software (Mediso Medical Imaging Systems, Budapest, Hungary). Volumes of interest (VOIs) for the primary tumor and metastatic lymph nodes were segmented with different methods: automatic and manual delineation using a fixed low threshold value of SUV >2.5 and fixed percent relative threshold (40%) of SUVmax, as these are commonly used according to literature [12]. Finally, data extraction was executed from manually segmented VOIs with fixed low threshold, since this method seemed to include the most of the real tumor volume according to anatomical informations on CT. (Figure 1) Furthermore, care was taken to exclude physiologic or inflammatory regions with high FDG uptake. Metabolic tumor volume (MTV) was calculated separately for the primary tumor, nodal metastases, and total tumor burden. Total lesion glycolysis (TLG) was also computed by multiplying MTV by the corresponding SUVmean, providing a surrogate for glucose metabolism. Due to the limitations of partial volume effect in small lesions, patients with a primary tumor <2 cm in size were excluded from the metabolic analysis.

### Statistical analysis

By using visual analysis, measure of skewness and statistical tests (Kolmogorov–Smirnov, Shapiro–Wilk) indicated that most variables deviate from normal distribution. (Exceptions: age at diagnosis, staging SUVmax of tumor plus node and tumor alone.) Consequently, we used Spearman rank order correlations as non-parametric method. For survival analysis, uni- and multivariate Cox-regressions, Kaplan-Meier method and Chi2-tests were used. Overall survival was measured in weeks. Further we applied logistic regression to detect the factors contributing to complete remission following full dose irradiation (at least 50 Gy). Significance level was set at *p*<0.05. All statistical analyses were performed using TIBCO Statistica 14.0.

## Results

### Basic characteristics

A total of 60 patients were enrolled in the study. The mean age at diagnosis was 60.6 years (mean 62.7 in p16-negative and 58.2 in p16-positive OPSCC subgroup). Based on immunohistochemical analysis, 28 patients were classified as p16-positive and 32 as p16-negative. Among the p16-positive patients, HPV DNA testing identified 24 HPV DNA-positive, 2 HPV DNA-negative, and 2 nonvaluable cases. PD-L1 expression was analyzed according to p16 status. Among p16-negative patients, 6% had TPS >50%, 75% had CPS >1, and 22% had CPS >20. In the p16-positive group, 18% had TPS >50%, 79% had CPS >1, and 32% had CPS >20. More detailed patient characteristics are listed in Table 1.

**Table 1:** Descriptive statistics

		Total	p16 negative	p16 positive
Total patient number		60 (100%)	32 (53%)	28 (47%)
HPV DNA (within p16-positive cases)	negative			2 (7%)
	positive			24 (86%)
	unknown			2 (7%)
Gender	male	32 (53%)	11 (34%)	21 (75%)
	female	28 (47%)	21 (66%)	7 (25%)
Clinical stage	I	13 (22%)	1 (3%)	12 (43%)
	II	13 (22%)	3 (9%)	10 (36%)
	III	9 (15%)	4 (13%)	5 (18%)
	IV	24 (40%)	23 (72%)	1 (4%)
	unknown	1 (2%)	1 (3%)	0 (0%)
T stage	1	4 (7%)	2 (6%)	2 (7%)
	2	22 (37%)	7 (22%)	15 (54%)
	3	12 (20%)	4 (13%)	8 (29%)
	4	22 (37%)	19 (59%)	3 (11%)
	unknown	0 (0%)	0 (0%)	0 (0%)
ECOG status	0	26 (44%)	12 (38%)	14 (50%)
	1	15 (25%)	7 (22%)	8 (29%)
	2	2 (3%)	2 (6%)	0 (0%)
	3	3 (5%)	3 (9%)	0 (0%)
	unknown	14 (23%)	8 (25%)	6 (21%)
Smoking status	Never	14 (23%)	2 (6%)	12 (43%)
	Former	6 (10%)	2 (6%)	4 (14%)
	Current	29 (48%)	24 (75%)	5 (18%)
	unknown	11 (18%)	4 (13%)	7 (25%)
Alcohol consumption	Never/rarely	25 (42%)	10 (31%)	15 (54%)
	Former	6 (10%)	6 (19%)	0 (0%)
	Current	17 (28%)	12 (38%)	5 (18%)
	unknown	12 (20%)	4 (13%)	8 (29%)
TPS	< 50%	53 (88%)	30 (94%)	23 (82%)
	≥ 50%	7 (12%)	2 (6%)	5 (18%)
CPS	< 1	14 (23%)	8 (25%)	6 (21%)
	≥ 1	46 (77%)	24 (75%)	22 (79%)
	< 20	44 (73%)	25 (78%)	19 (68%)
	≥ 20	16 (27%)	7 (22%)	9 (32%)
Age at diagnosis	x < 50	11 (18%)	2 (6%)	9 (32%)
	50 ≤ x < 60	15 (25%)	8 (25%)	7 (25%)
	60 ≤ x < 70	27 (45%)	19 (59%)	8 (29%)
	70 ≤ x < 80	6 (10%)	3 (9%)	3 (11%)
	80 ≤ x	1 (2%)	0 (0%)	1 (4%)
Remission following full dose irradiation	no	5 (8%)	4 (13%)	1 (4%)
	yes	22 (37%)	8 (25%)	14 (50%)
	criteria not met	33 (55%)	20 (63%)	13 (46%)

Eastern Cooperative Oncology Group Performance Status (ECOG), Tumor Proportion Score (TPS), Combined Positive Score (CPS)

### Correlation between metabolic PET/CT parameters and PD-L1 expression

In the subgroup of patients with HPV DNA-positive OPSCC, statistically significant positive correlations were identified between metabolic PET/CT parameters and PD-L1 expression. Specifically, total lesion glycolysis of the primary tumor and nodal regions (STAGING TLG T+N; Spearman’s ρ = 0.501) and metabolic tumor volume (STAGING MTV T+N; Spearman’s ρ = 0.512) positively correlated with the Tumor Proportion Score (TPS). When considering cases with CPS >20, these correlations remained positive, with corresponding Spearman coefficients of 0.441 and 0.491, respectively.

### Survival analysis

Univariate Cox regression analysis demonstrated that total lesion glycolysis (TLG) of the primary tumor was significantly associated with shorter survival in the whole cohort (*p*= 0.001, HR (hazard ratio) = 1.002) and even among the subgroups (p16-negative: *p*=0.041, HR=1.001, p16-positive: *p*= 0.011, HR= 1.003). Multivariate Cox regression (Table 2) revealed the strong prognostic effect of TLG, independent of p16, MTV and even primary tumor stage following TNM8.

**Table 2:** Multivariate Cox regression of TLG and MTV (primary tumor), p16 and tumor TNM8 stage. (Test Chi2 = 14.011, df = 4, *p* = 0.007, *N* = 60)

	Beta	Standard	Beta 95% lower	Beta 95% upper	t-value	Wald	*p*	Risk ratio	Risk ratio 95% lower	Risk ratio 95% upper
TLG primary tumor (g)	0.004	0.002	0.000	0.008	2.041	4.167	0.041	1.004	1.000	1.008
MTV primary tumor (ml, threshold: 2.5)	−0.027	0.020	−0.066	0.011	−1.397	1.950	0.163	0.973	0.937	1.011
p16 0/1 (negative/positive)	−0.618	0.451	−1.502	0.266	−1.370	1.876	0.171	0.539	0.223	1.305
Tumor stage	0.305	0.218	−0.123	0.734	1.397	1.953	0.162	1.357	0.884	2.082

Total lesion glycolysis (TLG), metabolic tumor volume (MTV)

**Fig. 2:**
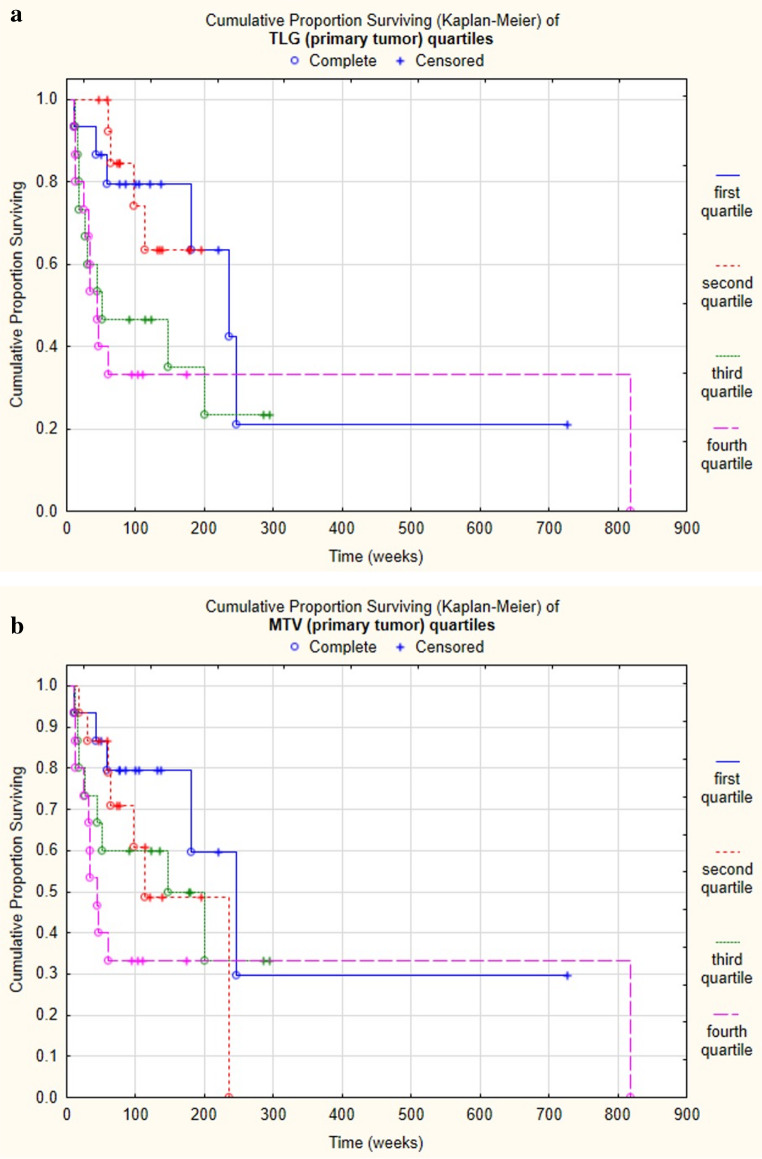
**A**: Cumulative Proportion Surviving (Kaplan-Meier) of TLG (primary tumor) quartiles on the whole OPSCC population. Chi-square test proved significantly different survival between groups. First quartile: TLG< 80.2275 g. Second quartile: 80.228 g ≤ TLG < 124.975 g. Third quartile: 124.975 g ≤. TLG < 280.206 g. Fourth quartile: 280.206 g ≤ TLG (Chi2 = 12.440, df = 3, *p* = 0.006, *N*=60). **B**: Cumulative Proportion Surviving (Kaplan-Meier) of MTV (primary tumor) quartiles on the whole OPSCC population. Chi-square test could not prove significant difference in survival between groups. First quartile: MTV< 15.62 ml. Second quartile: 15.62 ml ≤ MTV < 20.62 ml. Third quartile: 20.62 ml ≤ MTV < 39.38 ml. Fourth quartile: 39.38 ml ≤ MTV (Chi2 = 7.499, df = 3, *p* = 0.058, *N*=60)

Metabolic tumor volume (MTV) of the primary tumor appeared to be a less marked prognostic factor, as it did affect survival in the whole group as a sole marker (univariate Cox regression: *p*=0.005, HR= 1.012) and in p16-positive subgroup (*p*=0.003 h= 1.037), but was not significant in the p16-negative subgroup (*p*= 0.185) and in the multivariate Cox regression analysis (Table 2). We performed Kaplan-Meier analysis with the different quartiles on the whole population, which showed significant difference only in case of TLG (primary tumor, *p* = 0.006) highlighting its superiority (Figure 2 A and B). Other PET/CT parameters did not impact survival significantly.

The log-rank test demonstrated that p16-positive patients have significantly longer survival compared to p16-negative cases (*p*=0.038; RR (risk ratio) =0.431), which aligns with the well-established favorable prognosis of HPV-associated oropharyngeal cancer. Similarly, HPV DNA-positivity was associated with improved survival outcomes (*p*=0.005; RR=0.133).

### Association between PET/CT parameters and response to chemoradiation

Using logistic regression analysis, each PET/CT metabolic parameter was tested individually along with clinical stage to account for potential confounding effect. Staging MTV of the primary tumor emerged as a significant independent predictor of complete response to chemoradiotherapy (*p* = 0.026; OR (odds ratio) =1.08, Table 3). Importantly, patients with lower MTV values had substantially higher odds of achieving complete remission, regardless of tumor stage. Other parameters (SUV [max], TLG of primary tumor alone and with neck metastasis and MTV of primary tumor with neck metastasis) did not show significant prognostic effect.

**Table 3:** Logistic regression, where complete remission following full dose irradiation refers to ’1’, residual disease for ’0’ in the model. Final loss: 6.504, Chi2 = 12.448, *p* = 0.002, *N* = 26)

	Const.B0	MTV primary tumor (ml, treshold 2.5)	Disease stage (TNM8)
Estimate	−7.750	0.076	1.198
Standard Error	3.710	0.034	0.812
t(23)	−2.089	2.230	1.475
p-value	0.048	0.036	0.154
−95%CI	−15.424	0.005	−0.482
+95%CI	−0.076	0.146	2.878
Wald’s Chi-square	4.364	4.973	2.175
p-value	0.037	0.026	0.140
Odds ratio (unit ch)	0.000	1.079	3.313
−95%CI	0.000	1.005	0.617
+95%CI	0.927	1.157	17.784
Odds ratio (range)		1012.909	36.378
−95%CI		1.650	0.235
+95%CI		621781.400	5624.772

Confidence interval (CI), metabolic tumor volume (MTV)

## Discussion

The present study adds evidence to the burden of recommendations that emphasize the role of FDG PET imaging as primary diagnostic workup in oropharyngeal cancers as total lesion glycolysis bears a prognostic potential and can facilitate identifying high risk disease (possibly not feasible to treatment de-escalation).

Moreover, in the era of immune-checkpoint inhibitors, exploring immune-related prognostic factors is of utmost importance, this work reveals a correlation between metabolic parameters (TLG, MTV) and tumor proportion score of PD-L1 expression among HPV-associated oropharyngeal cancers. Besides the importance in tumor biology, this finding might serve as possible predictive marker that needs to be investigated in the anti-PD1 therapy of HPV-related oropharyngeal tumors.

Recent systematic review revealed that PD-L1 expression may be a predictive marker for better overall survival in head and neck squamous cell carcinoma (HNSCC) patients treated with immune checkpoint-inhibitors [13] In parallel, metabolic FDG PET/CT parameters such as MTV and TLG are widely investigated prognostic factors in HNSCC. In view of these observations, and considering that cancer and peritumoral tissue metabolism may be associated with different FDG uptake on PET scans, we aimed to evaluate whether FDG metabolic parameters of the primary tumor are associated with PD-L1 expression levels and outcome measures in cases of oropharyngeal cancers.

A study conducted by Yun Chen et al. demonstrates that PD-L1/PD-L2 expression correlates with F-FDG PET textural parameters in HNSCC, suggesting metabolic heterogeneity on PET/CT imaging may reflect tumor immune activity. Tumors with higher PD-L1 expression exhibit distinct PET-derived metabolic patterns, reinforcing the link between tumor metabolism and immune evasion. This indicates that PET imaging could serve as a non-invasive tool for assessing PD-L1 status, aiding in immunotherapy selection [14].

Takahashi et al. found that MTV and TLG levels correlated with PD-L1 expression, although in oral cancer patients, these findings highlight the potential for integrating metabolic imaging and immunological markers, paving the way for personalized treatment strategies in the management of HNSCC [15].

In our study, the relationship between PET-derived metabolic parameters and PD-L1 expression in HPV DNA-positive OPSCC suggests a biologically meaningful association between tumor glycolytic activity and immune checkpoint upregulation. The observed positive correlations between volumetric PET/CT parameters— TLG and MTV—and PD-L1 expression (both TPS and CPS >20) may indicate a more active tumor microenvironment characterized by enhanced metabolic and immunological activity. This is consistent with previous findings that high metabolic tumor burden may drive immune escape via increased PD-L1 expression [6, 16] .These results support the potential role of PET/CT-derived volumetric biomarkers in non-invasive immune profiling of HPV-associated OPSCC.

Higher staging MTV and TLG were associated with worse survival in our cohort, which aligns with the established notion that increased tumor metabolic activity reflects more aggressive tumor biology. These findings are supported by a recent meta-analysis [17], which confirmed that elevated MTV and TLG independently predict poorer overall and disease-free survival in HNSCC. As suggested in the systematic review by Rijo-Cedeño, J., et al., these measurements are accessible with today’s technical facilities and metabolic parameters could be used to support clinical practice in stratifying risk groups. [5] This reinforces the prognostic value of PET/CT-derived volumetric parameters, particularly when considered alongside immune-related biomarkers such as PD-L1 expression and p16/HPV status.

In our study, a positive correlation between PET/CT metabolic parameters (MTV, TLG) and PD-L1 expression was observed both in the total cohort and in the p16-positive subgroup. Interestingly, this association was not seen in p16-negative cases, which may suggest different mechanisms regulating PD-L1 expression. While in HPV-associated OPSCC, PD-L1 upregulation may be linked to increased metabolic activity and adaptive immune responses, in p16-negative tumors, PD-L1 expression could be driven by alternative pathways.

Beyond the possible prognostic value, staging MTV of the primary tumor was also found to be a significant independent predictor of complete response to chemoradiotherapy. This finding is consistent with the results reported by Strongin et al. [18], who demonstrated that primary tumor volume is a significant prognostic factor in patients with advanced head and neck cancer undergoing definitive chemoradiotherapy. In a recent review focusing on oropharyngeal cancers, MTV and TLG values were found to predict (chemo)radiotherapy response in HPV-negative cases, while in HPV-positive tumors, data suggest that nodal metabolic parameters, 18F-FDG PET mid-treatment response assessment may better predict treatment outcomes [12]. Compared to our results, this suggests that metabolic tumor burden may influence treatment response, and therefore taking MTV value into considerations may help in the development of personalized treatment protocols.

The distinct microRNA expression profiles of HPV-positive and HPV-negative OPSCC, as described by Orosz et al., support the justification for personalized treatment strategies based on molecular characteristics. When integrated with metabolic and immunologic biomarkers—such as PET/CT parameters and PD-L1 expression—these molecular differences may help optimize treatment planning and follow-up [19].

These results emphasize the potential of PET-based biomarkers to complement tissue-based PD-L1 assessment, particularly when biopsies are limited or dynamic monitoring is required. Future research should validate these findings in larger cohorts and explore their predictive value for immune checkpoint inhibitor response.

Several limitations should be considered when interpreting our results. The retrospective, single-center design and relatively small sample size may limit the statistical power. On the other hand, variables that show significant differences between subgroups even with a smaller number of elements may have more significant biological relevance than those that only proved to be (independent) prognostic/predictive factors in a large cohort. Although the analysis focused exclusively on PD-L1 expression, this was justified by its clinical relevance and established role in everyday therapeutic decision-making, particularly in the context of immune checkpoint inhibitor therapy. Nevertheless, other immune-related biomarkers such as tumor-infiltrating lymphocytes (TILs) or tumor mutational burden (TMB), which may also influence prognosis and response to treatment, were not evaluated. Lastly, although metabolic tumor volumes were calculated using a fixed SUV threshold, it is important to acknowledge that no standardized segmentation protocol currently exists for PET/CT-based metabolic parameter analysis. Our preferred threshold of SUV > 2.5 is the most widely used [17]. This lack of consensus may affect reproducibility and comparability across studies.

## Conclusion

18F-FDG PET/CT metabolic parameters —particularly MTV and TLG—show meaningful associations with PD-L1 expression, survival outcomes, and response to chemoradiotherapy in OPSCC. The use of metabolic parameters, complemented with HPV, p16, PD-L1 status, would provide a promising opportunity for identifying high-risk patients and to plan treatment and follow-up. Integrating volumetric PET/CT data into routine clinical practice could support more informed, personalized decision-making, especially in the context of immunotherapy.
